# A new species of *Chiasmocleis* (Microhylidae, Gastrophryninae) from the Atlantic Forest of Espírito Santo State, Brazil

**DOI:** 10.3897/zookeys.428.7352

**Published:** 2014-07-24

**Authors:** João F. R. Tonini, Maurício C. Forlani, Rafael O. de Sá

**Affiliations:** 1 Department of Biology, University of Richmond, Richmond, VA 23173, USA; 2 Current Address: Department of Biological Sciences, The George Washington University, Washington, DC 20052, USA

**Keywords:** Amphibians, *Chiasmocleis quilombola* sp. n., cryptic species, phylogenetics, systematics

## Abstract

Among Neotropical microhylids, the genus *Chiasmocleis* is exceptionally diverse. Most species of *Chiasmocleis* were described in recent years based on external morphology, but recent studies using molecular data did not support the monophyly of the species groups clustered based on feet webbing. Furthermore, a phylogeographic study of *C. lacrimae* estimated high genetic divergence and low gene flow among populations across small geographic ranges. Increasing the molecular and geographic sampling, and incorporating morphological data, we identified new cryptic species. Herein, we used novel genetic and morphological data to describe a new species of *Chiasmocleis*.

## Introduction

The diversity of evolutionary lineages with little phenotypic differences (i.e., cryptic species) might be better understood in the light of genetic delimitation of evolutionary units (Thomé et al. 2012, Hambäck et al. 2013). Recent molecular phylogenies of anuran, including work on species from the Brazilian Atlantic Forest, did not recovered as monophyletic the species groups clustered based mostly on morphology (Amaro et al. 2009, Canedo and Haddad 2012, Fouquet et al. 2012, Thomé et al. 2012).

Species are segments of population level evolutionary lineages and do not necessarily need to be phenetically distinguishable, diagnosable, monophyletic, intrinsically reproductively isolated, ecologically divergent, or anything else to be considered species, but they only have to be evolving separately from other lineages (de Queiroz 1998, de Queiroz 2007).

A recent molecular phylogeny (de Sá et al. 2012) recovered a polyphyletic *Chiasmocleis* and, to render the genus monophyletic, transferred one species to *Elachistocleis* and three species to *Syncope*. Recently, Peloso et al. (2014) placed *Syncope* in the synonymy of *Chiasmocleis*. *Chiasmocleis* is the most diverse genus of Neotropical microhylids, with 29 species distributed throughout Amazonia, Atlantic Forest, and open areas in South America, such as the Brazilian Cerrado and the Chaco of Bolivia and Paraguay (Cruz et al. 1997, de Sá et al. 2012, Peloso et al. 2014).

Tonini et al. (2013) in a phylogeographic analysis estimated high genetic divergence and low gene flow among populations of *Chiasmocleis lacrimae* (described as *Chiasmocleis carvalhoi* Cruz et al. 1997) in the Brazilian Atlantic Forest. Samples of two potential new species and of “*Chiasmocleis capixaba*” with less feet webbing were included as populations of *Chiasmocleis lacrimae*. Moreover, the study suggested that populations isolated-by-distance could represent recently diversified species, estimated to Miocene and Pliocene. Increasing the sampling along the distribution of *Chiasmocleis lacrimae* and *Chiasmocleis capixaba* and using additional molecular and morphological data, we were able to differentiate the phylogenetic structure and morphological differences associate to intraspecific and interspecific variation. We found that *Chiasmocleis lacrimae* and *Chiasmocleis capixaba* were not recovered as monophyletic, in fact populations corresponding to undescribed distinct evolutionary lineages. Although these undescribed lineages have similar body size and shape, and low nuclear divergence, they are exceptionally divergence in mitochondrial markers and are geographically structured.

Herein, we describe a new species of *Chiasmocleis* from the Atlantic Forest of southeastern Brazil and present a phylogenetic hypothesis for the species group.

## Material and methods

**Figure 1.**
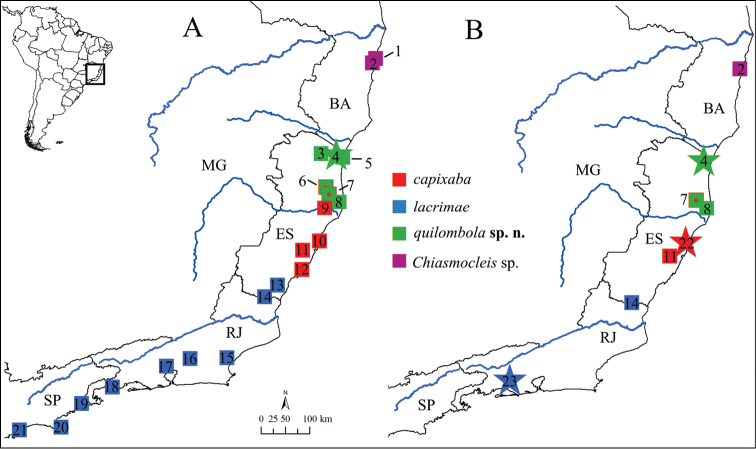
Sample localities of **A** tissues and **B** specimens included in the present study. Sites with more than one color indicates syntopy. List of localities: **1** Porto Seguro, **2** Trancoso, **3** ReBio Córrego Veado, **4** FloNa do Rio Preto (type locality of *Chiasmocleis quilombola* sp. n.), **5** Parque Estadual de Itaúnas, **6** ReBio Sooretama, **7** Reserva Natural Vale, **8** Povoação, **9** FloNa dos Goytacazes, **10** Costa Bela, **11** ReBio Duas Bocas, **12** Guarapari, **13** Mata da Usina Paineiras, **14** Mimoso do Sul, **15** ReBio União, **16** Cachoeiras de Macacu, **17** Duque de Caxias, **18** Angra dos Reis, **19** Picinguaba, **20** Ilha de São Sebastião, **21** Bertioga, **22** Aracruz (type locality of *Chiasmocleis capixaba*), **23** Horto Florestal (type locality of *Chiasmocleis lacrimae*). Blue lines represent major coastal rivers, from North to South: Jequitinhonha, Mucuri, Doce, and Paraíba do Sul. BA = Bahia State, ES = Espírito Santo State, RJ = Rio de Janeiro State, SP = São Paulo State, and MG = Minas Gerais State.

Specimens and tissues used herein and comparative material are deposited in the following collections: 1) CFBH: Coleção de Anfíbios Célio Fernando Baptista Haddad, Departamento de Zoologia, Universidade Estadual Paulista Rio Claro, Rio Claro, São Paulo State, Brazil; 2) MNRJ: Museu Nacional do Rio de Janeiro, Rio de Janeiro, Rio de Janeiro State, Brazil; 3) Museu de Zoologia, Universidade de São Paulo, São Paulo, São Paulo State, Brazil; 4) MBML: Museu de Biologia Mello Leitão, Santa Teresa, Espírito Santo State, Brazil; 5) CTA: Coleção de Tecidos e DNA da Universidade Federal do Espírito Santo (UFES) and LGA: Laboratório de Genética Animal, Vitória, Espírito Santo State, Brazil; 6) RN and CTRN: Universidade Federal Rural do Rio de Janeiro, Seropédica, Rio de Janeiro State, Brazil. Field numbers correspond to M. T. Rodrigues (MTR), Universidade de São Paulo, São Paulo, São Paulo State, Brazil; P. Rocha (PEU), Universidade Federal da Bahia, Salvador, Bahia State, Brazil; and J. F. R. Tonini (JFRT), vouchers are at UFES. Specimens examined and tissues samples are listed in Appendix 1 and 2, respectively, and sample localities are shown in Figure 1.

The following measurements were adapted from Duellman (2001) and Peloso and Sturaro (2008); measurements were taken for 56 individuals with a digital caliper under a stereomicroscope to the nearest 0.1 mm: SVL (snout-vent length); HDL (hand length; from the base of the thenar tubercle to the tip of the third finger); HDL4 (hand length from the base of the thenar tubercle to the tip of the fourth finger); HL (head length; from snout to angle of the jaw); HW (head width; between the angle of jaws); ED (eye diameter; between anterior and posterior corner of the eye); IOD (inter-orbital distance; distance between anterior corner of the eyes); IND (inter-nostril distance); END (eye-nostril distance; from the anterior corner of the eye to the posterior margin of nostril); THL (thigh length; from the center of the cloaca opening to the outer edge of the flexed knee); TBL (tibia length; from the outer edge of the flexed knee to the heel); FAL (forearm length); FL (foot length; from tibia-tarsal articulation to tip of fourth toe); 3FD (diameter of third finger disk); 4TD (diameter of fourth toe disk). Fingers and toes are numbered and abbreviated as follows: Fingers I–IV = FI–IV, Toes I–V = TI–V.

*Molecular Analyses*: Total genomic DNA was extracted from ethanol-preserved liver or muscle tissues using Qiagen DNeasy kit (Valencia, California, USA). We used four molecular markers (mtDNA: 12S, 16S, and NADH dehydrogenase subunit 2 [ND2]; nucDNA: brain-derived neurotrophic factor [BDNF]), amplified using previously published primer sets and PCR profiles (de Sá et al. 2012, Tonini et al. 2013). We performed a multiple loci alignment using an iterative procedure to compute a series of alignment/tree pairs in SATé-II (Liu et al. 2012), using default settings. GenBank accession numbers are given in Appendix 2.

The following outgroup were chosen based on published phylogenies including species of *Chiasmocleis* (de Sá et al. 2012): *Chiasmocleis leucosticta*, *Chiasmocleis mantiqueira*, *Chiasmocleis crucis*, *Chiasmocleis schubarti*, and *Chiasmocleis cordeiroi*. We selected a total of 100 samples (ingroup includes 69 samples) for a data set consisting of 2,473 base pairs. The best partition schemes and substitution models (Table 1) were chosen using PARTITION FINDER v1.1.1 (Lanfear et al. 2012) and used in phylogenetic analysis downstream.

**Table 1.** Best partition scheme and substitution models selected using Partition Finder.

Subset	Best Model	Subset partitions	Subset sites
1	HKY+I+G	12S	1–700
2	HKY+G	16S, ND2_1	701–1044, 1661–2473\3
3	K80+I	BDNF	1045–1660
4	HKY+G	ND2_2	1662–2473\3
5	GTR+G	ND2_3	1663–2473\3

We applied two approaches of phylogenetic estimation: 1) Maximum Likelihood (ML) and 2) Bayesian inference (BI) using the dataset containing the markers 12S, 16S, ND2, and BDNF. Maximum Likelihood in RAXML v7.2.8 (Stamatakis 2006) used a rapid-bootstrap with 1000 replications. Bayesian Inference in BEAST v1.7.4 (Drummond et al. 2012) used birth-death process as tree prior, linked tree models across partition, relaxed clock model with linked mitochondrial markers, but not the nuclear gene. The BI analysis ran for 50 million generations and the parameters were sampled every 5,000 generations producing a total of 10,000 trees. We discarded the first 1,000 trees as burnin in TREEANOTATOR. The output file was checked using TRACER v1.5 and values of Effective Sample Size >200 were considered suitable. Nodes having bootstrap values >70 in ML and posterior probabilities >0.95 in BI were considered as well supported. Analyses were performed through Cipres (Miller et al. 2010) and trees were visualized and edited using FIGTREE. Data available from the Dryad Digital Repository: http:// 10.5061/dryad.gm41t. Genetic distance (p-uncorrected) was calculated in MEGA5.0 (Tamura et al. 2011). A second species awaits description (Forlani et al. *submitted*) and it is referred throughout this manuscript as *Chiasmocleis* sp.

## Results

**Figure 2.**
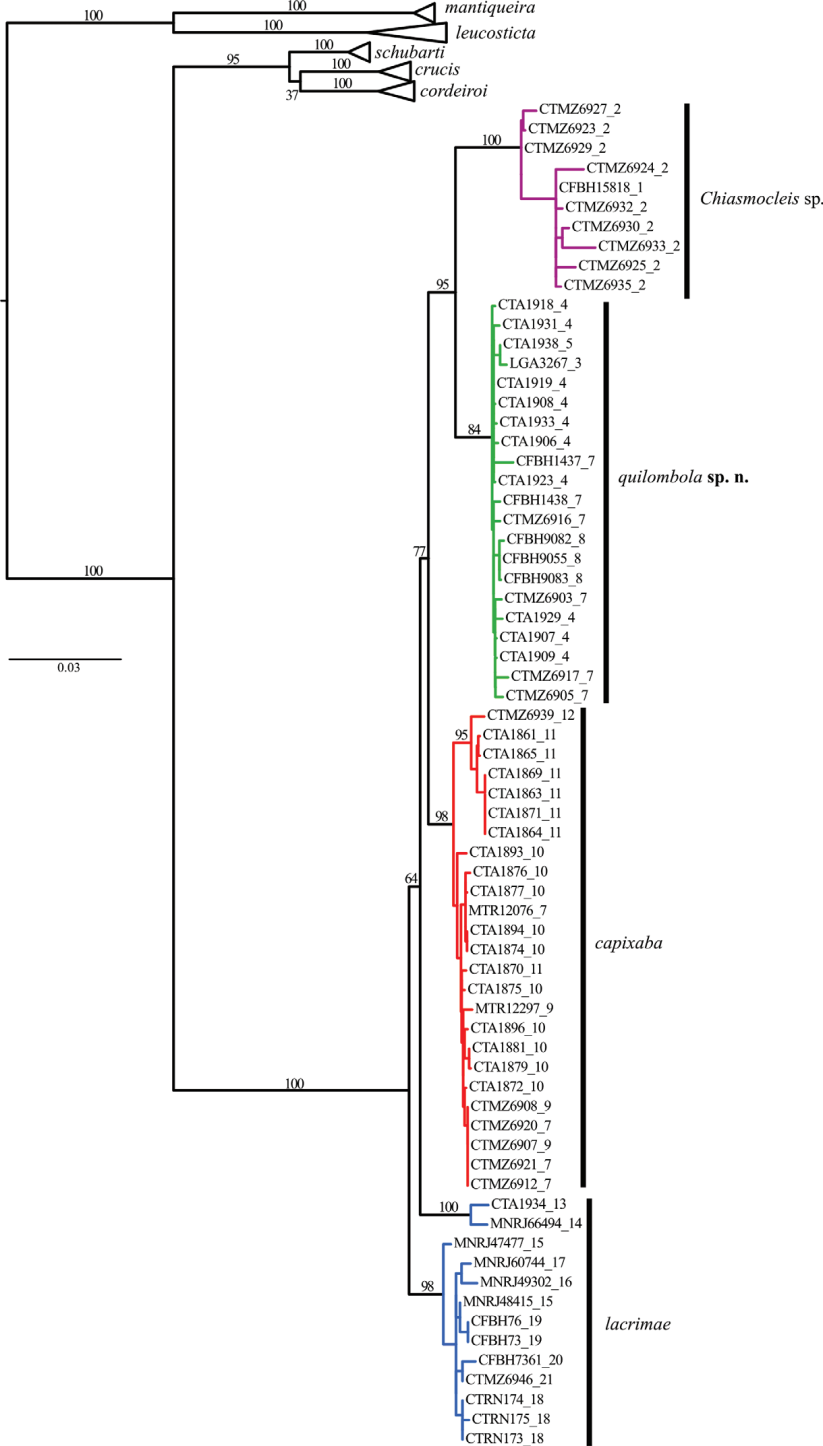
Maximum likelihood tree including 12S, 16S, ND2, and BDNF. Node numbers correspond to bootstrap, values >70 indicate good support. Although *Chiasmocleis lacrimae* may not represent a monophyletic species, bootstrap values are low to make further assumptions. Numbers after underscore symbol correspond to localities present in Figure 1. Scale bar represents number of substitutions/site.

**Figure 3.**
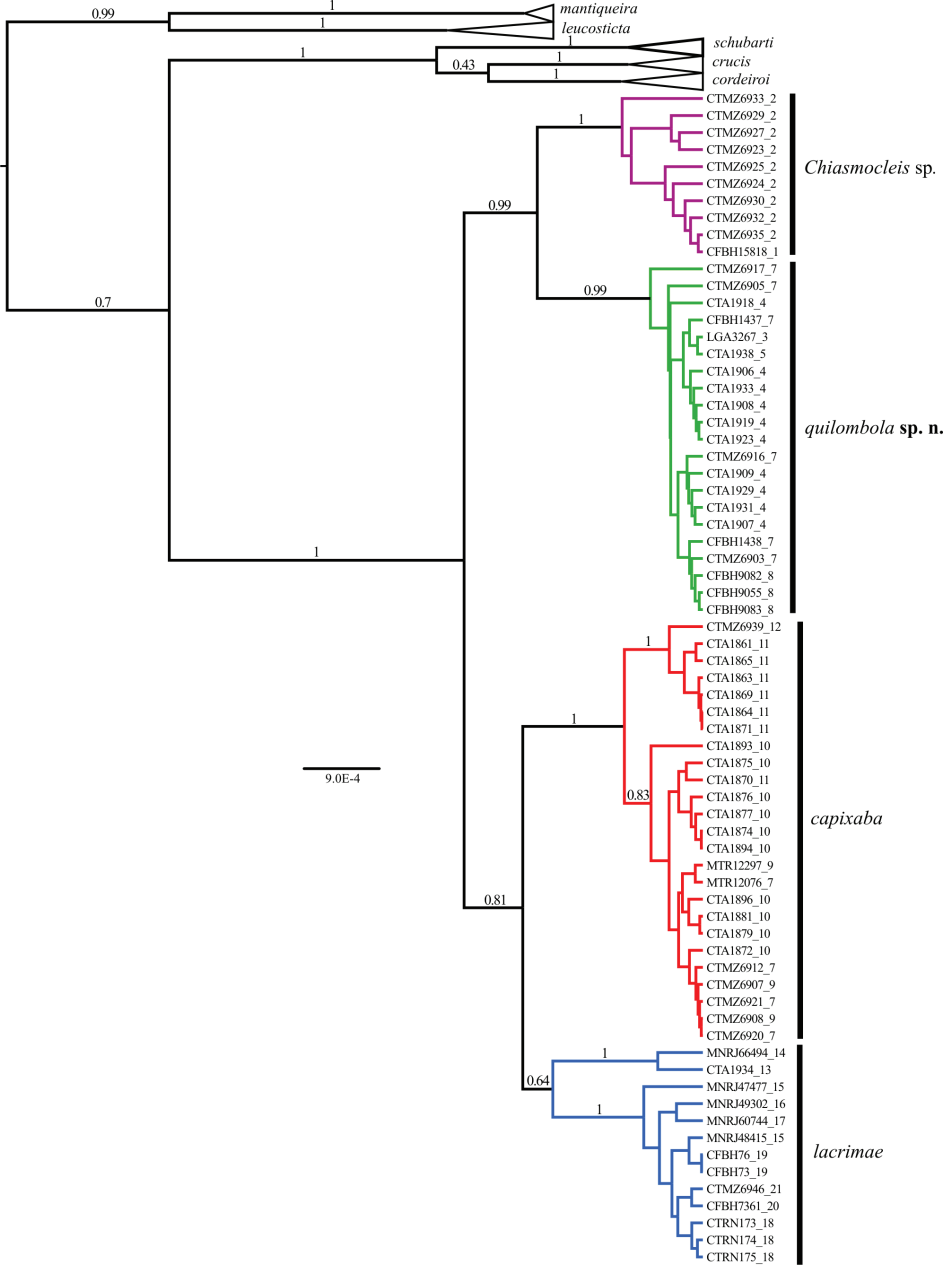
Phylogenetic hypothesis obtained through Bayesian Inference using 12S, 16S, ND2, and BDNF. Node numbers correspond to posterior probabilities, values >0.95 indicate good support. Numbers after underscore symbol correspond to localities present in Figure 1. Scale bar represents number of substitutions/site.

The phylogenetic hypotheses generated through ML (Figure 2) and the BI (Figure 3) resulted in similar topology. The ML tree (Figure 2) supported two new species as sister group of *Chiasmocleis capixaba*, *Chiasmocleis lacrimae* correspond to a basal node; whereas in the BI tree (Figure 3) *Chiasmocleis capixaba* was estimated as sister to *Chiasmocleis lacrimae*, but the posterior probability of this node was lower than 0.95. Both the ML and BI trees showed clades of *Chiasmocleis leucosticta*, *Chiasmocleis mantiqueira*, *Chiasmocleis crucis*, *Chiasmocleis schubarti*, and *Chiasmocleis cordeiroi*, but not *Chiasmocleis lacrimae* and *Chiasmocleis capixaba* (Figure 2, 3). Populations of *Chiasmocleis capixaba* and *Chiasmocleis lacrimae* from the north of the Espírito Santo State, as well as populations of *Chiasmocleis lacrimae* from southern areas of the Bahia State, would represent two new cryptic lineages closely related to *Chiasmocleis lacrimae* and *Chiasmocleis capixaba*. In the ML analysis populations from southern Espírito Santo formed a clade that makes *Chiasmocleis lacrimae* polyphyletic (Figure 2), whereas in the BI these populations formed a clade including also populations of *Chiasmocleis lacrimae* from the states of São Paulo and Rio de Janeiro (Figure 3). However, given the lack of support for this node in both analyses, basing taxonomic change on the presumed polyphyly is not warranted at present.

Therefore, our results show that the new species clusters within the genus *Chiasmocleis*.

## Description of a new species

### Chiasmocleis quilombola sp. n.

Taxon classificationAnimaliaAnuraMicrohylidae

http://zoobank.org/81CD38A6-72C6-4CAF-A4AC-45C011459A0E

Figure 4

#### Holotype.

**Figure 4.**
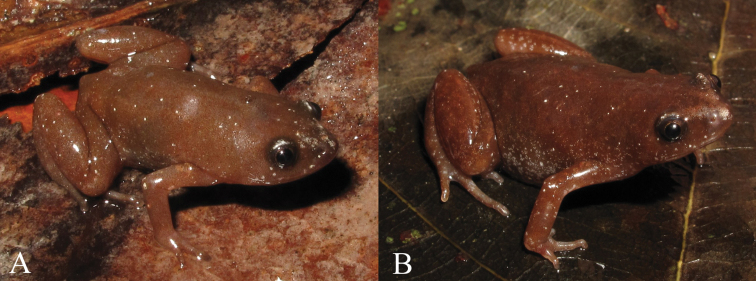
*Chiasmocleis quilombola* sp. n. *in vivo*. **A** male (holotype: MZUSP147478) and **B** female (MZUSP147479, paratopotype). Not in scale.

MZUSP147478, adult male, collected at the Floresta Nacional do Rio Preto (Figure 4A), Municipality of Conceição da Barra, Espírito Santo State, Brazil (18°21'19"S, 39°50'39"W), collected on December 8-16, 2009, by L. P. Costa, J. F. R. Tonini, J. Dalapicolla, R. Duda, and C. M. Mattedi.

#### Paratopotypes.

Males: MZUSP147471–73, MZUSP147475–76, MZUSP147494; female: MZUSP147479 (Figure 4B), Municipality of Conceição da Barra, Espírito Santo State, Brazil (18°21'19"S, 39°50'39"W), collected on December 8-16, 2009, by L. P. Costa, J. F. R. Tonini, J. Dalapicolla, R. Duda, and C. M. Mattedi.

#### Diagnosis.

A small-sized species of *Chiasmocleis* (males SVL mean= 14 ± 1.4 mm; female SVL = 17.1 mm), diagnosed by the following combination of characters: (1) body slender; (2) snout rounded in lateral and dorsal views; (3) all fingers slightly fringed, not webbed, in males and female; (4) all toes fringed and slightly webbed in males and female; (5) dermal spines on fingers and toes of males can be present or absent, absent in female; (6) dermal spines on dorsal surface of males can be present or absent, absent in female; (7) dermal spines absent on ventral surface in males and female; (8) dermal spines on chin and snout of males can be present or absent, absent in female; (9) dermal spines over outer surfaces of legs and cloaca in males can be present or absent, absent in female; (10) female has para-cloacal glands; (11) incomplete occipital fold; (12) vocal slits present in males; (13) dorsal coloration brown; (14) medial ventral body surface light cream colored, whereas ventrolateral surfaces have a light brown and cream marbled pattern; (15) ventral surfaces of fore and hind limbs with a homogeneously and finely dark pattern over a cream background; (16) dorsal surface of fore and hind limbs light brown with a few cream spots or blotches, more distinct on the fore limbs; (17) male throat infuscate; (18) mid-dorsal and/or line on posterior surface of thighs may be present; and (19) tympanum indistinct.

#### Description of holotype.

**Figure 5.**
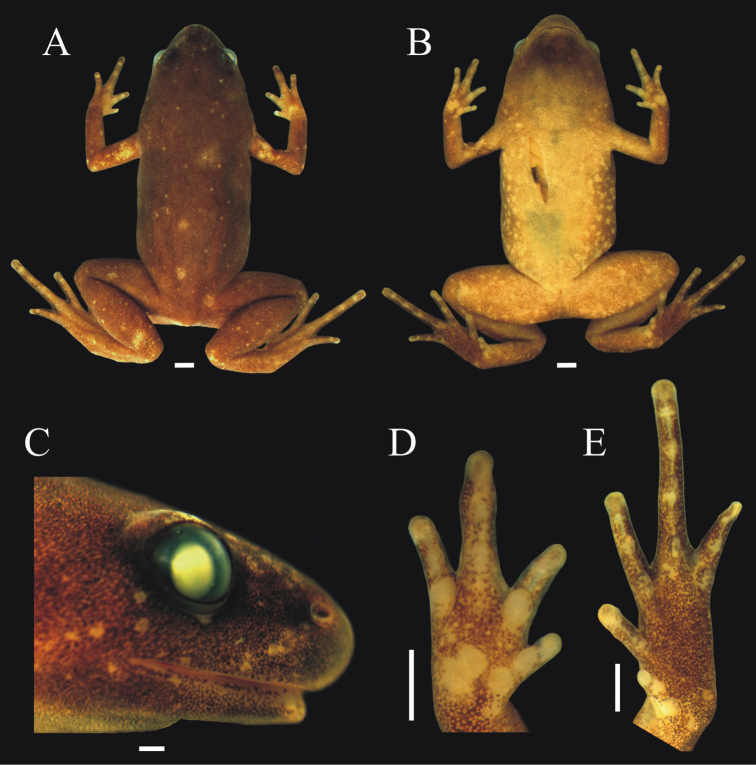
Holotype of *Chiasmocleis quilombola* sp. n. (MZUSP 147478). **A** Dorsal **B** ventral, and **C** lateral views **D** right hand and **E** right foot. White bars = 1 mm.

Body small (SVL = 15.7 mm), slender, slightly ovoid (Figure 5); head triangular in shape, broader than long; snout short, tip of snout rounded (Figure 5A–B); nostrils located closer to the tip of snout than to eye, not protuberant, directed laterally (Figure 5C); inter-nostril distance smaller than eye–nostril distance and smaller than eye diameter; canthus rostralis slightly defined; loreal region slightly convex; lips not flared; eyes small, slightly protruding; inter-orbital area flat; incomplete occipital fold; tympanum indistinct; upper jaw projecting beyond lower one; tongue large, elongate, and laterally free; premaxillae, maxillae, and vomerine teeth absent; choanae small, rounded, widely separated, positioned anterolaterally to eye; vocal slit present.

Arms slender, lacking tubercles on forearm. Hands not webbed (Figure 5D); fingers tips rounded, not expanded, and slightly fringed; fingers lacking dermal spines; finger lengths I<II<IV<III; thumb without nuptial asperities; subarticular tubercles well developed and rounded, proximal subarticular tubercles larger than others; supernumerary tubercles absent; thenar tubercle well developed, ovoid, and at the base of finger I; two palmar tubercles, a rounded inner tubercle and an elongated outer one (Figure 5D). Legs short, moderately robust; knee and heel lacking tubercles; tibial and tarsal ridges absent. Foot slightly webbed (Figure 5A–B, E); toes slightly fringed; toe tip rounded lacking disks; subarticular tubercles well developed, ovoid; supernumerary tubercles absent; an oval inner, but no outer, metatarsal tubercle. Toe lengths I<II<V<III<IV; toes lacking dermal spines; tibia length slightly shorter than thigh length; combined thigh and tibia lengths approximately 82.8% of snout-vent length; foot length approximately 43.9% of snout-vent length.

Skin smooth, dorsal surfaces of body lacking dermal spines. Throat black and few dermal spines found on chin and snout (Figure 5B). Cloaca lacks para-cloacal tubercles or glands.

#### Coloration in preservative.

Dorsum dark brown with a few small cream spots and blotches; dorsal surface of limbs dark brown with cream blotches and small spots, particularly on the proximal forelimb; palm of hands marbled brown and pale cream, foot dark brown; belly surface cream, dorsolateral and ventral surfaces with a marbled pale brown and cream pattern; throat dark brown to black. Ventral surface of thighs light brown with a finely reticulated dark pattern over a cream background cream with a few cream spots more evident close to the edges; ventral surfaces of tibia and tarsus finely marbled in light brown with cream, lighter than the dorsal surface. Absence of distinct lines on the body and limbs.

#### Measurements of holotype

(in mm). SVL 15.7; HDL 3.4; HDL4 2.3; HL 2.7; HW 4.5; ED 1.3; IOD 2.8; IND 1.1; END 1.2; THL 6.5; TBL 6.4; FL 6.9, FAL 3.2; 3FD 0.3; 4TD 0.4.

#### Variation in the type series.

Measurements data of the type series are given in Table 2 and information of the comparative material are provided in Appendix 1. Overall, the type series agrees with the holotype coloration; one specimen has a mid-dorsal line and a line on the posterior surface of the thighs and also more dermal spines (MZUSP147475). The incomplete occipital fold varied from indistinct to weakly visible laterally (= incomplete). The combined mean thigh and tibia length represents approximately 81% of mean snout-vent length in males, and 77.7% in females; foot length approximately 41.6% of snout-vent length in males and 39.7% in females.

**Table 2.** Morphometric measurements (mm) of the type series of *Chiasmocleis quilombola* sp. n.

Specimen	Type	Sex	SVL	HL	HW	ED	IOD	IND	END	THL	TBL	FL	3FD	4TD	FAL	HDL	HDL4
MZUSP147471	Paratype	Male	14.4	2.8	3.8	1.0	2.6	1.1	1.5	6.1	6.3	6.2	0.3	0.3	3.0	2.8	1.8
MZUSP147472	Paratype	Male	15.3	2.8	4.1	1.3	2.7	1.2	1.3	7.1	6.6	6.6	0.3	0.4	3.2	3.4	2.2
MZUSP147473	Paratype	Male	16.1	3.2	3.9	1.1	2.2	1.1	1.3	6.0	6.0	6.4	0.2	0.2	3.2	3.3	2.1
MZUSP147475	Paratype	Male	14.5	2.8	4.6	1.3	2.6	1.3	1.3	6.6	6.4	6.7	0.4	0.4	3.3	3.5	2.2
MZUSP147476	Paratype	Male	16.1	2.7	4.4	1.2	2.7	1.3	1.4	6.7	6.3	6.1	0.3	0.5	3.0	3.3	1.8
MZUSP147478	Holotype	Male	15.7	2.8	4.5	1.3	2.8	1.1	1.3	6.6	6.5	6.9	0.4	0.4	3.2	3.5	2.3
MZUSP147494	Paratype	Male	16.6	3.1	4.6	1.4	2.6	1.2	1.4	7.0	6.9	7.6	0.3	0.4	3.3	3.8	2.4
MZUSP147479	Paratype	Female	17.2	2.9	4.6	1.3	2.9	1.3	1.4	6.7	6.6	6.8	0.3	0.4	3.3	3.5	2.3

Abbreviations: SVL = snout-vent length; HDL = hand length; HDL4 = hand length from the base of the thenar tubercle to the tip of the fourth finger; HL = head length; HW = head width; ED = eye diameter; IOD = inter-orbital distance; IND = inter-nostril distance; END = eye-nostril distance; THL = thigh length; TBL = tibia length; FAL = forearm length; FL = foot length; 3FD = diameter of third finger disk; 4TD = diameter of fourth toe disk. For tissues numbers see Appendix 2.

#### Etymology.

The specific epithet *quilombola* refers to people who inhabit quilombo communities. Historically, quilombos were communities constituted by and used as refuges for escaped slaves between 1530 and 1815 during colonial Portuguese rule in Brazil. Nowadays in the north of Espírito Santo Estate quilombola communities still remain and maintain alive their traditions, such as quilombola food and craftwork. This species' name is indeclinable.

#### Distribution.

*Chiasmocleis quilombola* sp. n. is known from localities between the Doce River and the Mucuri River, e.g., Floresta Nacional do Rio Preto and Parque Estadual de Itaúnas, Municipality of Conceição da Barra; Reserva Biológica Córrego Veado, Municipality of Pinheiros; Reserva Natural, Reserva Biológica de Sooretama, and Cocoa plantations in Povoação, Municipality of Linhares (Appendix 2). The populations assigned to *Chiasmocleis lacrimae* and *Chiasmocleis capixaba* at northernmost of Espírito Santo State are allocated to the new taxon *Chiasmocleis quilombola* sp. n. (Figure 1).

#### Natural history.

**Figure 6.**
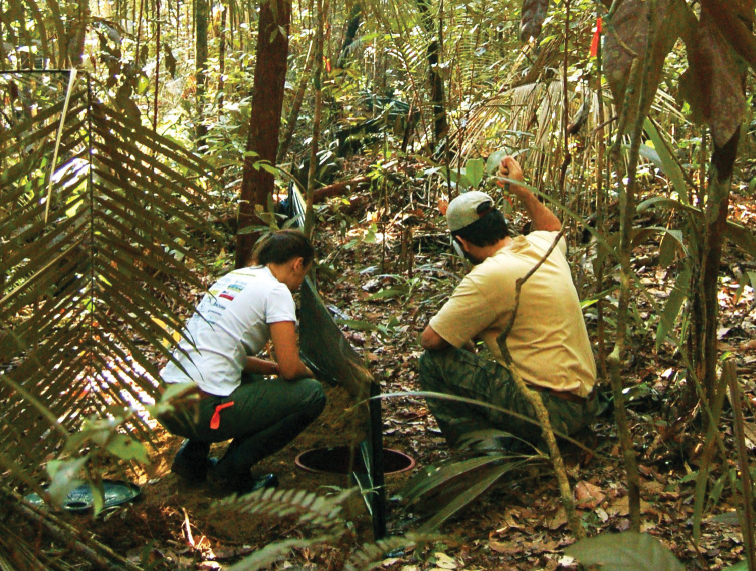
Larissa Gaigher and Dr. Yuri Leite inspecting pitfall traps installed at the type locality of *Chiasmocleis quilombola* sp. n., Floresta Nacional do Rio Preto, Municipality of Conceição da Barra, Espírito Santo State, Brazil.

*Chiasmocleis quilombola* sp. n. was collected in pitfall traps after heavy rains at Floresta Nacional do Rio Preto (Figure 6). The lines of pitfalls were installed at the vicinity of a permanent lagoon and a temporary swamp. The Floresta Nacional do Rio Preto has 2,830 ha and an elevation between five to 50 m above the sea level. The soil is typical of coastal areas, mostly sand. The area consists of secondary forested areas and plantations with few remnants of primary Atlantic Forest.

## Discussion

*Chiasmocleis quilombola* sp. n. has been misidentified as *Chiasmocleis lacrimae* and *Chiasmocleis capixaba* due to an overlap in feet webbing and body size (e.g., Tonini et al. 2013, see below). *Chiasmocleis quilombola* sp. n. corresponds to clade N2, *Chiasmocleis* sp. to clade N1, *Chiasmocleis capixaba* to central clade, and *Chiasmocleis lacrimae* to southern clades of Tonini et al. (2013). Our morphological observations and comparisons with other species combined with molecular information support *Chiasmocleis quilombola* sp. n. and *Chiasmocleis* sp. as separate evolutionary lineages (see below and Figure 1, 2). Low levels of genetic divergence in the BDNF (Table 3) between *Chiasmocleis quilombola* sp. n. and closely related species is consistent with recent cladogenetic events and supports a previous study that estimated initial speciation within this clade (i.e., *Chiasmocleis lacrimae*, *Chiasmocleis capixaba*, *Chiasmocleis quilombola* sp. n., and *Chiasmocleis* sp.) during the Miocene/Pliocene (Tonini et al. 2013). *Chiasmocleis quilombola* sp. n. and *Chiasmocleis* sp. corresponds to an earlier lineage split dated to approximately the Pliocene/Pleistocene (Tonini et al. 2013).

**Table 3.** Genetic distance (p-uncorrected) in the BDNF (upper-right) and in the ND2 (lower-left) among *Chiasmocleis quilombola* sp. n. and sister species. Values at the diagonal correspond to the genetic distance within species in the ND2.

Species	*Chiasmocleis capixaba*	*Chiasmocleis lacrimae*	*Chiasmocleis cordeiroi*	*Chiasmocleis crucis*	*Chiasmocleis quilombola* sp. n.	*Chiasmocleis schubarti*	*Chiasmocleis* sp.
*Chiasmocleis capixaba*	**0.015**	0.001	0.008	0.008	0.001	0.007	0.003
*Chiasmocleis lacrimae*	0.064	**0.044**	0.008	0.008	0.001	0.007	0.003
*Chiasmocleis cordeiroi*	0.211	0.227	**0.013**	0.005	0.008	0.004	0.007
*Chiasmocleis crucis*	0.182	0.198	0.108	**0.015**	0.008	0.005	0.007
*Chiasmocleis quilombola* sp. n.	0.071	0.082	0.217	0.209	**0.006**	0.007	0.004
*Chiasmocleis schubarti*	0.206	0.204	0.107	0.103	0.22	**0.017**	0.007
*Chiasmocleis* sp.	0.098	0.104	0.253	0.237	0.083	0.236	**0.023**

*Chiasmocleis quilombola* sp. n. is distinct from *Chiasmocleis schubarti* (species with which occurs in sympatry) in having smaller snout-vent length, feet slightly webbed, cream ventral surface, and marbled light brown and cream dorsolateral pattern instead of larger snout-vent length, absence of feet webbing and belly pattern roughly marbled in dark brown and light cream in *Chiasmocleis schubarti* (Cruz et al. 1997). *Chiasmocleis quilombola* sp. n. is most similar to *Chiasmocleis lacrimae* and *Chiasmocleis capixaba*, species with which it has been previously confused (e.g. Tonini et al. 2013). However, the new species is distinguished from closely relatives by the following set of characters: 1) a smaller body size, shorter head length, shorter thigh and tibia compared to *Chiasmocleis lacrimae*, *Chiasmocleis capixaba*, and *Chiasmocleis* sp. (Table 4), 2) smaller eye diameter, inter-orbital distance, inter-nostril distance, diameter of third finger disk, and diameter of fourth toe disk than *Chiasmocleis capixaba*, 3) smaller eye-nostril distance, feet length, hand length, and hand length to the tip of the fourth finger than *Chiasmocleis lacrimae*. Moreover, males of *Chiasmocleis quilombola* sp. n. have less webbing on the foot (more extensive web on the foot in *Chiasmocleis capixaba*, absent in *Chiasmocleis* sp., and ranging from little to absent in *Chiasmocleis lacrimae*; Cruz et al. 1997, Peloso et al. 2014, Forlani et al. *submitted*). The new species has slender arms, legs, finger, and toes (robust arms and legs in *Chiasmocleis lacrimae*; thick fingers and toes in *Chiasmocleis capixaba*), as well as smaller and less abundant dermal spines in males (spines larger and abundant in *Chiasmocleis lacrimae*; abundant in *Chiasmocleis capixaba*). Males of *Chiasmocleis quilombola* sp. n. posses less amount of fringes between fingers II and III and a slender third finger than males *Chiasmocleis capixaba*.

**Table 4.** Differences between *Chiasmocleis capixaba*, *Chiasmocleis lacrimae*, *Chiasmocleis quilombola* sp. n., and *Chiasmocleis* sp.

Species	*Chiasmocleis capixaba*	*Chiasmocleis lacrimae*	*Chiasmocleis quilombola* sp. n.	*Chiasmocleis* sp.
*Chiasmocleis capixaba*	SVL=15.1 (SD 0.6) HL=2.8 (SD 0.1) THL=6.2 (SD 0.3) TBL=6.1 (SD 0.3)	Feet webbing	head length; thickness of limbs, fingers, and toes; dermal spines	Feet webbing; thickness of limbs; dermal spines
*Chiasmocleis lacrimae*	mtDNA (ND2: 6.4%, 16S: 1.3%, 12S: 1.8%) nuDNA (BNDF: haplotype sharing)	SVL=16.1 (SD 0.9) HL=3.3 (SD 0.2) THL=6.6 (SD 0.4) TBL=6.5 (SD 0.3)	head and limb length; feet webbing; thickness of limbs; dermal spines	body size; thickness; dermal spines
*Chiasmocleis quilombola* sp. n.	mtDNA (ND2: 7.1%, 16S: 0.8%, 12S: 1.1%) nuDNA (BNDF: haplotype sharing)	mtDNA (ND2: 8.2%, 16S: 0.8%, 12S: 1.8%) nuDNA (BNDF: haplotype sharing)	SVL=14 (SD 1.4) HL=2.6 (SD 0.2) THL=5.8 (SD 0.7) TBL=5.6 (SD 0.6)	Feet webbing; thickness of limbs
*Chiasmocleis* sp.	mtDNA (ND2: 9.8%, 16S: 2.2%, 12S: 1.2%) nuDNA (BNDF: no haplotype sharing)	mtDNA (ND2: 10%, 16S: 2.3%, 12S: 2.1%) nuDNA (BNDF: no haplotype sharing)	mtDNA (ND2: 8.3%, 16S: 1.6%, 12S: 2.1%) nuDNA (BNDF: haplotype sharing)	SVL=15.3 (SD 0.7) HL=3.4 (SD 0.1) THL=6 (SD 0.3) TBL=6.2 (SD 0.3)

SVL snout-vent length; HL head length; THL tight length; TBL tibia length; SD standard deviation.

*Chiasmocleis quilombola* sp. n. are distinguished from other *Chiasmocleis* species by: 1) four externally evident fingers and five toes distinguishes it from *Chiasmocleis antenori*, *Chiasmocleis carvalhoi*, and *Chiasmocleis tridactyla* (digit reduction; Walker 1973, Nelson 1975, Duellman and Mendelson 1995); 2) a shorter snout-vent length differentiate it from *Chiasmocleis alagoanus*, *Chiasmocleis albopunctata*, *Chiasmocleis anatipes*, *Chiasmocleis atlantica*, *Chiasmocleis avilapiresae*, *Chiasmocleis bassleri*, *Chiasmocleis centralis*, *Chiasmocleis cordeiroi*, *Chiasmocleis crucis*, *Chiasmocleis devriesi*, *Chiasmocleis hudsoni*, *Chiasmocleis leucosticta*, *Chiasmocleis magnova*, *Chiasmocleis mehelyi*, *Chiasmocleis papachibe*, *Chiasmocleis royi*, *Chiasmocleis sapiranga*, *Chiasmocleis shudikarensis*, *Chiasmocleis supercilialba*, and *Chiasmocleis ventrimaculata* (larger snout-vent length; Dunn 1949, Bokermann 1952, Walker and Duellman 1974, Caramaschi and Pimenta 2003, Cruz et al. 1997, Cruz et al. 1999, Caramaschi and Cruz 1997, Cruz et al. 2007a, Moravec and Köhler 2007, Peloso and Sturaro 2008, Funk and Cannatella 2009, Morales and McDiarmid 2009, Peloso et al. 2014); 3) small feet webbing of males and females distinguish the new species from *Chiasmocleis cordeiroi*, *Chiasmocleis leucosticta*, *Chiasmocleis mantiqueira*, and *Chiasmocleis sapiranga* (more extensive webbed feet in males and females; Cruz et al. 1997, Cruz et al. 2007a, b); 4) a light cream belly pattern without dark spots distinguished it from *Chiasmocleis alagoanus*, C*. atlantica*, *Chiasmocleis haddadi*, *Chiasmocleis leucosticta*, and *Chiasmocleis mantiqueira* (belly pattern roughly marbled in dark brown and pale cream, Cruz et al.1997, Cruz et al.1999, Cruz et al. 2007b, Peloso et al. 2014); and 5) snout rounded and belly light cream colored differentiate it from *Chiasmocleis gnoma* (snout truncate and belly boldly marbled in brown and pale cream; Canedo et al. 2004).

*Chiasmocleis quilombola* sp. n. occurs in sympatry with *Chiasmocleis schubarti* at the Floresta Nacional do Rio Preto, Municipality of Conceição da Barra, and at the Reserva Biológica Córrego Veado, Municipality of Pinheiros; it also occurs with *Chiasmocleis capixaba* and *Chiasmocleis schubarti* at the Reserva Natural Vale, Reserva Biológica de Sooretama, and at Cocoa plantations in Povoação, sites in the Municipality of Linhares. The new species is allopatric to *Chiasmocleis* sp. and *Chiasmocleis lacrimae* (Figure 1). We did not have access to tissues samples of *Chiasmocleis capixaba* from Nova Viçosa, Bahia State (Van Sluys 1998), to include in the genetic analysis, thus the phylogenetic relationship of this population remains unclear.

Cryptic species have challenged our ability to assess current levels of biodiversity. Anuran taxonomy has used various data sources to describe the species diversity, e.g., advertisement calls, external morphology, osteology, tadpoles, ecology, molecular data, karyotypes (Duellman and Trueb 1986, Haas 2003). However, *Chiasmocleis* systematics has been based on external morphology from adults and behavioral information (Wogel et al. 2004, Hartmann et al. 2002, Nascimento and Skuk 2006, Oliveira Filho and Giaretta 2006, Langone et al. 2007, Peloso and Sturaro 2008, Rodrigues et al. 2008, Santana et al. 2012, but see Peloso et al. 2014). Sexual dimorphism in size, amount of webbing, and color pattern have been useful characters to diagnose species (Cruz et al. 1997). Recent molecular studies 1) demonstrated the non-monophyly of traditionally recognized species groups (de Sá et al. 2012, Peloso et al. 2014) and 2) reported high genetic divergences and low gene flow along small geographical scales, suggesting that some populations could represent new species (Tonini et al. 2013). Given the current overall biodiversity crisis and specifically the worldwide threats to amphibian biodiversity, molecular studies should move beyond the identification of genetic clades and should make every effort to formally describe those evolutionary lineages. Herein, we have taken this approach and described a new species based on a combination of morphological characters in a clade of cryptic species with shown high genetic diversity and low gene flow.

The new species occupy coastal areas North of Espírito Santo State, a region that is under strong human pressure. Therefore, marine and coastal communities are susceptible to impacts of proposed modifications in the landscape for the exploitation of mineral resources. In this context, *Chiasmocleis quilombola* sp. n. may face imminent threat of habitat loss, as consequence of the deforestation and intensive occupation of the space by human activities.

## Supplementary Material

XML Treatment for Chiasmocleis quilombola

## Appendix 1

**Table 5.** Examined material and measurements from males included in the morphological analyses.

***Chiasmocleis***	**Numbers**	**Locality**	**State**	**SVL**	**HL**	**HW**	**ED**	IOD	IND	END	THL	TBL	FL	3FD	4TD	FAL	HDL	HDL4
*Chiasmocleis capixaba*	MNRJ17514	Aracruz	ES	14.77	2.91	4.04	1.18	2.57	1.16	1.21	6.19	6.34	6.43	0.35	0.44	2.98	3.36	2.08
*Chiasmocleis capixaba*	MNRJ17515	Aracruz	ES	13.98	2.84	4.44	1.28	2.71	1.21	1.39	6.17	6.13	6.58	0.29	0.46	3.07	3.28	1.94
*Chiasmocleis capixaba*	MNRJ17516	Aracruz	ES	14.47	2.93	3.98	1.18	2.64	1.19	1.32	6.08	5.96	6.64	0.30	0.42	3.10	3.36	2.16
*Chiasmocleis capixaba*	MNRJ17517	Aracruz	ES	14.70	3.00	4.10	1.36	2.61	1.29	1.26	6.23	6.10	6.74	0.33	0.51	3.15	3.55	2.32
*Chiasmocleis capixaba*	MNRJ17518	Aracruz	ES	16.02	3.13	4.39	1.32	2.64	1.21	1.45	6.61	6.80	7.45	0.38	0.48	3.62	3.86	2.33
*Chiasmocleis capixaba*	MNRJ17519	Aracruz	ES	15.40	2.99	3.95	1.36	2.63	1.23	1.56	6.05	6.31	6.28	0.33	0.45	3.38	3.17	2.09
*Chiasmocleis capixaba*	MNRJ17520	Aracruz	ES	14.94	2.76	3.82	1.26	2.66	1.20	1.36	5.71	6.06	5.90	0.41	0.47	2.96	3.15	1.80
*Chiasmocleis capixaba*	MNRJ17535	Aracruz	ES	15.23	2.96	3.83	0.97	2.39	1.18	1.07	6.18	5.76	5.97	0.38	0.42	2.76	3.05	2.13
*Chiasmocleis capixaba*	MNRJ17536	Aracruz	ES	15.24	2.50	3.92	1.23	2.60	1.16	1.41	5.83	5.79	6.63	0.33	0.45	2.93	3.52	2.19
*Chiasmocleis capixaba*	MNRJ17895	Aracruz	ES	15.46	2.89	4.32	1.12	2.56	1.22	1.21	6.62	6.30	6.46	0.33	0.47	3.25	3.34	2.15
*Chiasmocleis capixaba*	MNRJ22962	Reserva Natural Vale	ES	14.83	3.02	4.44	1.26	2.67	1.18	1.38	6.29	6.08	6.37	0.36	0.44	3.12	3.34	2.08
*Chiasmocleis capixaba*	MNRJ22966	Reserva Natural Vale	ES	15.05	2.99	4.41	1.37	2.71	1.19	1.43	6.71	6.20	6.25	0.36	0.52	3.18	3.15	1.72
*Chiasmocleis capixaba*	MZUSP147468	ReBio Duas Bocas	ES	15.88	3.05	4.40	1.27	2.87	1.25	1.41	6.84	6.53	6.92	0.43	0.43	3.06	3.44	2.14
*Chiasmocleis capixaba*	MZUSP147469	ReBio Duas Bocas	ES	16.23	3.02	4.61	1.05	2.99	1.31	1.68	6.80	6.71	7.05	0.48	0.48	3.28	3.29	2.30
*Chiasmocleis lacrimae*	MNRJ17480	Horto Florestal	RJ	16.20	3.40	4.40	1.10	2.70	0.90	1.40	6.50	6.50	7.30	0.30	0.40	3.30	3.60	2.50
*Chiasmocleis lacrimae*	MNRJ17481	Horto Florestal	RJ	15.60	3.30	4.10	1.10	2.60	1.00	1.40	6.20	6.30	7.20	0.40	0.40	3.50	3.60	2.60
*Chiasmocleis lacrimae*	MNRJ17482	Horto Florestal	RJ	16.20	3.50	2.60	1.30	2.60	1.00	1.90	6.50	6.50	7.10	0.40	0.50	3.30	3.80	2.50
*Chiasmocleis lacrimae*	MNRJ17484	Horto Florestal	RJ	19.15	3.57	4.82	1.35	2.84	1.44	1.69	7.45	7.35	8.23	0.34	0.36	3.56	4.23	2.69
*Chiasmocleis lacrimae*	MNRJ17485	Horto Florestal	RJ	16.10	3.70	4.30	1.20	2.50	0.90	1.60	6.90	6.70	7.50	0.30	0.40	3.40	3.60	2.40
*Chiasmocleis lacrimae*	MNRJ17486	Horto Florestal	RJ	15.07	3.00	4.33	1.22	2.52	1.22	1.50	6.30	6.22	6.68	0.30	0.43	3.19	3.44	2.24
*Chiasmocleis lacrimae*	MNRJ17487	Horto Florestal	RJ	16.10	3.33	4.38	1.19	2.91	1.30	1.68	6.94	6.78	7.16	0.27	0.39	3.46	3.53	2.08
*Chiasmocleis lacrimae*	MNRJ17488	Horto Florestal	RJ	15.90	3.20	3.80	1.10	2.50	0.90	1.30	5.80	6.20	6.60	0.30	0.40	3.00	3.30	2.20
*Chiasmocleis lacrimae*	MNRJ17489	Horto Florestal	RJ	15.40	3.10	3.90	1.20	2.20	0.90	1.30	6.10	6.10	6.90	0.30	0.40	3.10	3.40	2.10
*Chiasmocleis lacrimae*	MNRJ17490	Horto Florestal	RJ	16.10	3.30	4.00	1.10	2.50	0.90	1.40	6.70	7.10	7.70	0.40	0.40	3.50	4.20	2.70
*Chiasmocleis lacrimae*	MNRJ17491	Horto Florestal	RJ	15.26	2.90	4.75	1.21	2.92	1.47	1.49	6.59	6.46	7.13	0.32	0.41	3.34	3.78	2.41
*Chiasmocleis lacrimae*	MNRJ17492	Horto Florestal	RJ	15.50	3.90	4.60	1.30	2.50	1.20	1.50	6.10	6.30	6.50	0.40	0.40	3.10	3.70	2.50
*Chiasmocleis lacrimae*	MNRJ17498	Horto Florestal	RJ	17.10	3.70	4.70	1.20	2.50	1.10	1.40	6.70	6.80	6.90	0.40	0.50	3.40	3.40	2.50
*Chiasmocleis lacrimae*	MNRJ17505	Horto Florestal	RJ	16.80	3.42	4.62	1.41	2.84	1.32	1.71	7.19	6.88	7.43	0.33	0.38	3.50	3.87	2.42
*Chiasmocleis lacrimae*	MNRJ17506	Horto Florestal	RJ	16.95	3.30	4.84	1.36	3.00	1.35	1.62	7.19	6.80	7.32	0.41	0.43	3.60	4.09	2.63
*Chiasmocleis lacrimae*	MNRJ17507	Horto Florestal	RJ	15.16	3.14	4.43	1.29	2.71	1.24	1.45	6.06	6.13	6.28	0.34	0.41	3.12	3.51	2.22
*Chiasmocleis lacrimae*	MNRJ17565	Horto Florestal	RJ	16.66	2.97	4.42	1.36	2.79	1.30	1.47	7.05	6.69	7.14	0.49	0.43	3.23	3.97	2.63
*Chiasmocleis lacrimae*	MNRJ66497	Mimoso do Sul	ES	16.60	3.14	4.77	1.40	2.89	1.31	1.48	6.69	6.49	6.90	0.39	0.43	3.55	3.80	2.32
*Chiasmocleis quilombola* sp. n.	MNRJ29057	Povoação	ES	12.72	2.70	3.89	1.05	2.42	1.08	1.20	5.35	5.05	5.24	0.30	0.39	2.65	2.79	1.79
*Chiasmocleis quilombola* sp. n.	MNRJ29058	Povoação	ES	13.23	2.68	3.90	1.12	2.47	1.10	1.12	5.28	4.91	5.17	0.34	0.42	2.52	2.78	1.73
*Chiasmocleis quilombola* sp. n.	MNRJ29059	Povoação	ES	13.56	2.53	3.63	1.15	2.37	1.08	1.12	5.36	5.25	5.33	0.29	0.39	2.80	3.00	1.89
*Chiasmocleis quilombola* sp. n.	MNRJ29060	Povoação	ES	12.59	2.55	3.79	1.08	2.41	1.08	1.16	5.12	4.67	4.56	0.32	0.32	2.48	2.48	1.67
*Chiasmocleis quilombola* sp. n.	MNRJ29073	Povoação	ES	13.76	2.53	3.94	1.25	2.43	1.18	1.29	5.67	5.63	5.88	0.41	0.41	2.88	3.07	1.85
*Chiasmocleis quilombola* sp. n.	MNRJ29074	Povoação	ES	13.47	2.33	3.72	1.18	2.27	1.08	1.15	5.24	5.27	5.60	0.32	0.40	2.73	3.02	1.81
*Chiasmocleis quilombola* sp. n.	MBML2858	Povoação	ES	13.47	2.54	3.94	0.88	2.04	0.82	1.03	5.11	5.39	5.50	0.33	0.43	2.61	2.91	1.72
*Chiasmocleis quilombola* sp. n.	MBML2866	Povoação	ES	12.15	2.24	3.34	1.05	2.16	0.72	0.95	5.26	5.11	5.33	0.27	0.39	2.55	2.75	1.62
*Chiasmocleis quilombola* sp. n.	MBML2863	Povoação	ES	12.30	2.34	3.75	0.82	2.00	0.83	0.93	5.22	5.32	5.42	0.31	0.48	2.50	2.63	1.59
*Chiasmocleis quilombola* sp. n.	MZUSP147473	Flona Rio Preto	ES	16.07	3.18	3.87	1.09	2.18	1.09	1.33	6.01	6.00	6.41	0.24	0.23	3.15	3.30	2.07
*Chiasmocleis quilombola* sp. n.	MZUSP147478	Flona Rio Preto	ES	15.71	2.75	4.52	1.30	2.80	1.13	1.25	6.55	6.47	6.91	0.35	0.44	3.21	3.45	2.33
*Chiasmocleis quilombola* sp. n.	MZUSP147471	Flona Rio Preto	ES	14.43	2.75	3.83	1.03	2.57	1.11	1.51	6.07	6.27	6.23	0.25	0.26	2.99	2.84	1.83
*Chiasmocleis quilombola* sp. n.	MZUSP147475	Flona Rio Preto	ES	14.48	2.79	4.55	1.28	2.55	1.28	1.25	6.63	6.41	6.73	0.36	0.36	3.26	3.48	2.16
*Chiasmocleis quilombola* sp. n.	MZUSP147494	Flona Rio Preto	ES	16.55	3.14	4.63	1.36	2.55	1.24	1.36	7.02	6.89	7.59	0.25	0.37	3.33	3.80	2.40
*Chiasmocleis quilombola* sp. n.	MZUSP147476	Flona Rio Preto	ES	16.09	2.70	4.36	1.24	2.65	1.31	1.36	6.73	6.26	6.11	0.32	0.47	2.95	3.33	1.83
*Chiasmocleis quilombola* sp. n.	MZUSP147472	Flona Rio Preto	ES	15.28	2.80	4.08	1.33	2.67	1.24	1.33	7.08	6.55	6.60	0.34	0.41	3.20	3.40	2.22
sp.	MTR13495	Trancoso	BA	15.92	3.39	4.07	1.06	2.65	0.94	1.43	6.37	6.16	6.77	0.27	0.42	3.60	3.45	2.06
sp.	MTR13547	Trancoso	BA	13.80	3.53	4.09	0.97	2.36	1.00	1.35	5.59	5.68	5.72	0.27	0.35	2.95	3.18	2.27
sp.	MTR13545	Trancoso	BA	15.07	3.51	4.24	1.10	2.43	1.03	1.35	5.91	6.41	6.78	0.29	0.38	2.95	3.40	2.35
sp.	MTR13590	Trancoso	BA	15.48	3.48	3.99	1.17	2.51	0.80	1.32	6.01	6.22	6.69	0.30	0.45	3.11	3.73	2.41
sp.	MTR13565	Trancoso	BA	16.27	3.78	4.31	0.95	2.59	1.04	1.61	5.64	6.33	6.56	0.33	0.46	3.20	3.68	2.67
sp.	MTR13548	Trancoso	BA	15.36	3.34	4.57	1.06	2.52	1.12	1.09	6.44	6.62	6.72	0.30	0.40	3.19	3.64	2.36
sp.	MTR13546	Trancoso	BA	15.53	3.10	4.40	1.24	2.30	0.91	1.46	6.02	5.96	6.56	0.31	0.40	2.90	3.30	2.14
sp.	MTR13489	Trancoso	BA	15.34	3.53	4.05	1.16	2.35	1.00	1.36	6.67	6.54	6.73	0.27	0.44	3.40	3.46	2.40

## Appendix 2

**Table 6.** Samples included in the molecular analyses and genbank numbers. Numbers in bold were retrieved from previous studies.

***Chiasmocleis***	**Voucher**	**Tissue**	**Locality**	**State**	**12S**	**16S**	**ND2**	**BDNF**
*Chiasmocleis capixaba*	MZUSP147497	CTA1861	ReBio Duas Bocas	ES	KM111721	KM111817	**JQ410706**	KM111908
*Chiasmocleis capixaba*	MZUSP147498	CTA1863	ReBio Duas Bocas	ES	KM111722	KM111818	**JQ410707**	KM111909
*Chiasmocleis capixaba*	MZUSP147499	CTA1864	ReBio Duas Bocas	ES	KM111723	KM111819	**JQ410708**	KM111910
*Chiasmocleis capixaba*	MZUSP147468	CTA1865	ReBio Duas Bocas	ES	KM111724	KM111820	KM111992	KM111911
*Chiasmocleis capixaba*	MZUSP147482	CTA1869	ReBio Duas Bocas	ES	KM111725	KM111821	KM111993	KM111912
*Chiasmocleis capixaba*	MZUSP147469	CTA1870	ReBio Duas Bocas	ES	KM111726	KM111822	KM111994	KM111913
*Chiasmocleis capixaba*	MZUSP147500	CTA1871	ReBio Duas Bocas	ES	KM111727	KM111823	**JQ410709**	KM111914
*Chiasmocleis capixaba*	MZUSP147510	CTA1872	Serra	ES	KM111728	KM111824	**JQ410685**	KM111915
*Chiasmocleis capixaba*	MZUSP147512	CTA1874	Serra	ES	KM111729	KM111825	**JQ410690**	KM111916
*Chiasmocleis capixaba*	MZUSP147513	CTA1875	Serra	ES	KM111730	KM111826	**JQ410691**	KM111917
*Chiasmocleis capixaba*	JFT479	CTA1876	Serra	ES	KM111731	KM111827	**JQ410692**	KM111918
*Chiasmocleis capixaba*	MZUSP147514	CTA1877	Serra	ES	KM111732	KM111828	**JQ410693**	KM111919
*Chiasmocleis capixaba*	JFT483	CTA1879	Serra	ES	KM111733	KM111829	**JQ410694**	KM111920
*Chiasmocleis capixaba*	JFT499	CTA1881	Serra	ES	KM111734	KM111830	**JQ410695**	KM111921
*Chiasmocleis capixaba*	MZUSP147520	CTA1893	Serra	ES	KM111735	KM111831	**JQ410700**	KM111922
*Chiasmocleis capixaba*	MZUSP147521	CTA1894	Serra	ES	KM111736	KM111832	**JQ410701**	KM111923
*Chiasmocleis capixaba*	MZUSP147523	CTA1896	Serra	ES	KM111737	KM111833	**JQ410702**	KM111924
*Chiasmocleis capixaba*	MTR12276	CTMZ6907	FloNa dos Goytacazes	ES	KM111738	KM111834	-	KM111925
*Chiasmocleis capixaba*	MTR12296	CTMZ6908	FloNa dos Goytacazes	ES	KM111739	KM111835	**JQ410688**	KM111926
*Chiasmocleis capixaba*	MTR12407	CTMZ6912	Reserva Natural Vale	ES	KM111740	KM111836	-	-
*Chiasmocleis capixaba*	MTR12484	CTMZ6920	Reserva Natural Vale	ES	KM111741	KM111837	-	KM111927
*Chiasmocleis capixaba*	MTR12485	CTMZ6921	Reserva Natural Vale	ES	KM111742	KM111838	-	KM111928
*Chiasmocleis capixaba*	-	CTMZ6939	Guarapari	ES	KM111743	KM111839	-	KM111929
*Chiasmocleis capixaba*	MTR12076	MTR12076	Reserva Natural Vale	ES	KM111744	KM111840	**JQ410687**	KM111930
*Chiasmocleis capixaba*	MTR12297	MTR12297	FloNa dos Goytacazes	ES	KM111745	KM111841	**JQ410689**	KM111931
*Chiasmocleis cordeiroi*	CFBH32057	CFBH15784	Ilhéus	BA	KM111759	KM111852	KM111995	KM111939
*Chiasmocleis cordeiroi*	MZUSP147496	CTA1935	Ituberá	BA	KM111760	KM111853	KM111996	KM111940
*Chiasmocleis cordeiroi*	MTR22122	MTR22122	EE Wenceslau Guimarães	BA	KM111761	KM111854	KM111997	KM111941
*Chiasmocleis cordeiroi*	MTR22123	MTR22123	EE Wenceslau Guimarães	BA	KM111762	KM111855	KM111998	KM111942
*Chiasmocleis cordeiroi*	PEU137	PEU137	Jaguaripe	BA	KM111763	KM111856	KM111999	KM111943
*Chiasmocleis cordeiroi*	PEU146	PEU146	Jaguaripe	BA	KM111764	KM111857	KM112000	KM111944
*Chiasmocleis crucis*	MTR6001	CTMZ6898	Serra do Teimoso	BA	KM111765	KM111858	KM112001	KM111945
*Chiasmocleis crucis*	-	CTMZ6900	Ilhéus	BA	KM111766	KM111859	KM112002	KM111946
*Chiasmocleis crucis*	-	CTMZ6901	Ilhéus	BA	KM111767	KM111860	KM112003	KM111947
*Chiasmocleis crucis*	MTR16070	MTR16070	Serra Bonita	BA	KM111768	KM111861	KM112004	KM111948
*Chiasmocleis lacrimae*	-	CFBH73	Picinguaba	SP	KM111748	**KC180040**	**JQ410715**	**KC180202**
*Chiasmocleis lacrimae*	CFBH17495	CFBH7361	Ilha de São Sebastião	SP	KM111749	KM111844	-	
*Chiasmocleis lacrimae*	-	CFBH76	Picinguaba	SP	KM111750	**KC180063**	**JQ410714**	**KC180163**
*Chiasmocleis lacrimae*	JFT981	CTA1934	Matada Usina Paineiras	ES	KM111751	KM111845	**JQ410710**	KM111932
*Chiasmocleis lacrimae*	-	CTMZ6946	Bertioga	SP	KM111752	KM111846	-	KM111933
*Chiasmocleis lacrimae*	RN7003	CTRN173	Angra dos Reis	RJ	KM111753	KM111847	-	-
*Chiasmocleis lacrimae*	RN7004	CTRN174	Angra dos Reis	RJ	KM111754	KM111848	-	KM111934
*Chiasmocleis lacrimae*	RN7005	CTRN175	Angra dos Reis	RJ	KM111755	KM111849	-	KM111935
*Chiasmocleis lacrimae*	MNRJ47477	MNRJ47477	ReBio União	RJ	KM111746	KM111842	-	-
*Chiasmocleis lacrimae*	MNRJ48415	MNRJ48415	ReBio União	RJ	KM111747	KM111843	-	-
*Chiasmocleis lacrimae*	MNRJ49302	MNRJ49302	Cachoeiras de Macacu	ES	KM111756	KM111850	**JQ410712**	KM111936
*Chiasmocleis lacrimae*	MNRJ60744	MNRJ60744	Duque de Caxias	RJ	KM111757	-	**JQ410713**	KM111937
*Chiasmocleis lacrimae*	MNRJ66494	MNRJ66494	Mimoso do Sul	ES	KM111758	KM111851	**JQ410711**	KM111938
*Chiasmocleis leucosticta*	CFBH19029	CFBH8594	PE Ilha do Cardoso	SP	KM111769	KM111862	-	-
*Chiasmocleis leucosticta*	MZUSP136053	CTMZ2485	PE Carlos Botelho	SP	KM111770	KM111863	-	-
*Chiasmocleis leucosticta*	MZUSP136055	CTMZ2493	PE Carlos Botelho	SP	KM111771	-	-	KM111949
*Chiasmocleis leucosticta*	MZUSP136059	CTMZ2497	PE Carlos Botelho	SP	-	KM111864	-	KM111950
*Chiasmocleis leucosticta*	MTR7128	CTMZ6943	Fazenda Intervales	SP	-	-	KM112005	KM111951
*Chiasmocleis leucosticta*	-	CTMZ6944	Piedade	SP	KM111772	-	KM112006	-
*Chiasmocleis mantiqueira*	-	CTMZ6891	Serra do Brigadeiro	MG	KM111773	KM111865	-	KM111952
*Chiasmocleis mantiqueira*	UFMG-A9643	UFMG-T1802	Ouro Branco	MG	KM111774	KM111866	KM112007	-
*Chiasmocleis mantiqueira*	UFMG-A9659	UFMG-T1804	Ouro Branco	MG	KM111775	KM111867	-	KM111953
*Chiasmocleis mantiqueira*	UFMG-A9651	UFMG-T1810	Ouro Branco	MG	KM111776	KM111868	-	KM111954
*Chiasmocleis mantiqueira*	UFMG-A9656	UFMG-T1815	Ouro Branco	MG	KM111777	KM111869	-	KM111955
*Chiasmocleis quilombola* sp. n.	-	CFBH1437	ReBio Sooretama	ES	KM111778	**KC180044**	-	**KC180193**
*Chiasmocleis quilombola* sp. n.	-	CFBH1438	ReBio Sooretama	ES	KM111779	**KC179977**	-	**KC180168**
*Chiasmocleis quilombola* sp. n.	CFBH19471	CFBH9055	Povoação	ES	KM111780	KM111870	KM112008	KM111956
*Chiasmocleis quilombola* sp. n.	CFBH18076	CFBH9082	Povoação	ES	KM111781	KM111871	KM112009	KM111957
*Chiasmocleis quilombola* sp. n.	CFBH18077	CFBH9083	Povoação	ES	KM111782	KM111872	KM112010	KM111958
*Chiasmocleis quilombola* sp. n.	JFT831	CTA1906	FloNa do Rio Preto	ES	KM111783	KM111873	JQ410669	KM111959
*Chiasmocleis quilombola* sp. n.	MZUSP147471	CTA1907	FloNa do Rio Preto	ES	KM111784	KM111874	**JQ410670**	KM111960
*Chiasmocleis quilombola* sp. n.	MZUSP147472	CTA1908	FloNa do Rio Preto	ES	KM111785	KM111875	**JQ410671**	KM111961
*Chiasmocleis quilombola* sp. n.	MZUSP147473	CTA1909	FloNa do Rio Preto	ES	KM111786	KM111876	**JQ410672**	KM111962
*Chiasmocleis quilombola* sp. n.	MZUSP147474	CTA1918	FloNa do Rio Preto	ES	KM111787	KM111877	**JQ410673**	KM111963
*Chiasmocleis quilombola* sp. n.	MZUSP147475	CTA1919	FloNa do Rio Preto	ES	KM111788	KM111878	**JQ410674**	KM111964
*Chiasmocleis quilombola* sp. n.	MZUSP147494	CTA1923	FloNa do Rio Preto	ES	KM111789	KM111879	**JQ410677**	KM111965
*Chiasmocleis quilombola* sp. n.	MZUSP147479	CTA1929	FloNa do Rio Preto	ES	KM111790	KM111880	**JQ410679**	KM111966
*Chiasmocleis quilombola* sp. n.	MZUSP147480	CTA1931	FloNa do Rio Preto	ES	KM111791	KM111881	**JQ410680**	KM111967
*Chiasmocleis quilombola* sp. n.	MZUSP147493	CTA1933	FloNa do Rio Preto	ES	KM111792	KM111882	**JQ410681**	KM111968
*Chiasmocleis quilombola* sp. n.	JFT990	CTA1938	PE de Itaúnas	ES	-	KM111883	-	KM111969
*Chiasmocleis quilombola* sp. n.	MTR12017	CTMZ6903	Reserva Natural Vale	ES	KM111793	KM111884	-	KM111970
*Chiasmocleis quilombola* sp. n.	MTR12077	CTMZ6905	Reserva Natural Vale	ES	KM111794	KM111885	-	-
*Chiasmocleis quilombola* sp. n.	MTR12470	CTMZ6916	Reserva Natural Vale	ES	KM111795	KM111886	-	KM111971
*Chiasmocleis quilombola* sp. n.	MTR12471	CTMZ6917	Reserva Natural Vale	ES	KM111796	KM111887	-	KM111972
*Chiasmocleis quilombola* sp. n.	MTR21527	LGA3267	ReBio Córrego Veado	ES	KM111797	KM111888	**JQ410668**	KM111973
*Chiasmocleis schubarti*	CFBH9331	CFBH2078	ReBio Sooretama	ES	KM111798	**KC180071**	KM112011	**KC180122**
*Chiasmocleis schubarti*	CFBH18075	CFBH9060	Povoação	ES	KM111799	KM111889	KM112012	KM111974
*Chiasmocleis schubarti*	CFBH 22501	CTA1860	ReBio Duas Bocas	ES	KM111800	KM111890	KM112013	KM111975
*Chiasmocleis schubarti*	MZUSP147485	CTA1887	FloNa do Rio Preto	ES	KM111801	KM111891	KM112014	KM111976
*Chiasmocleis schubarti*	MZUSP147487	CTA1924	FloNa do Rio Preto	ES	KM111802	KM111892	KM112015	KM111977
*Chiasmocleis schubarti*	MTR12094	CTMZ6906	FloNa dos Goytacazes	ES	KM111803	KM111893	KM112016	KM111978
*Chiasmocleis schubarti*	LGA2630	LGA2630	ReBio Córrego Veado	ES	KM111804	KM111894	**JQ410661**	KM111979
*Chiasmocleis schubarti*	MTR12266	MTR12266	FloNa dos Goytacazes	ES	KM111805	KM111895	KM112017	KM111980
*Chiasmocleis schubarti*	MTR17524	MTR17524	PE do Rio Doce	MG	KM111806	KM111896	KM112018	KM111981
*Chiasmocleis schubarti*	MTR17571	MTR17571	PE do Rio Doce	MG	KM111807	KM111897	KM112019	KM111982
sp.	-	CFBH15818	Porto Seguro	BA	-	KM111898	-	KM111983
sp.	MTR13466	CTMZ6923	Trancoso	BA	KM111808	KM111899	**JQ410665**	KM111984
sp.	MTR13489	CTMZ6924	Trancoso	BA	KM111809	KM111900	**JQ410666**	KM111985
sp.	MTR13495	CTMZ6925	Trancoso	BA	KM111810	KM111901	-	KM111986
sp.	MTR13545	CTMZ6927	Trancoso	BA	KM111811	KM111902	-	KM111987
sp.	MTR13547	CTMZ6929	Trancoso	BA	KM111812	KM111903	-	KM111988
sp.	MTR13548	CTMZ6930	Trancoso	BA	KM111813	KM111904	-	KM111989
sp.	MTR13565	CTMZ6932	Trancoso	BA	KM111814	KM111905	-	KM111990
sp.	MTR13579	CTMZ6933	Trancoso	BA	KM111815	KM111906	-	KM111991
sp.	MTR13589	CTMZ6935	Trancoso	BA	KM111816	KM111907	KM112020	-
